# 

**DOI:** 10.1192/bjb.2024.110

**Published:** 2025-10

**Authors:** Nick Bass

**Affiliations:** Consultant psychiatrist and Director of Global Health, East London NHS Foundation Trust, London, UK. Email: nick.bass@nhs.net

**Figure:**
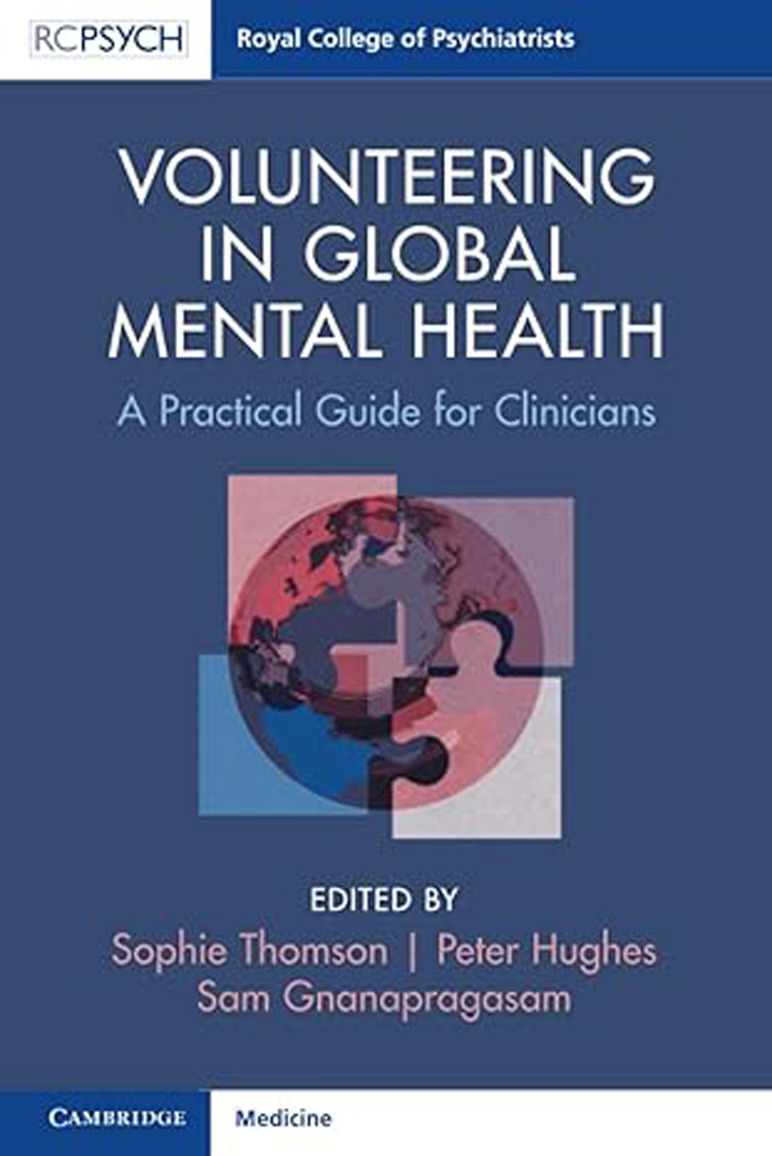

This deceptively short book is actually crammed with practical advice for anyone planning to volunteer in some capacity to support mental health development or delivery in the most resource-constrained parts of the world. The editors have extensive field experience and have added several contributions from others. There is a degree of geographical duplication as a result but even then, the same situation may be approached from different perspectives and this can still be valuable. As well as practical clinical and teaching tips, and discussion of practical issues for the novice in resource-poor countries, the real positives include attention to ethics, human rights and the need to work in partnership and some very helpful advice on how to cope on return to the home country (an overlooked issue for those who have experienced significant cultural displacement during their work abroad or have been volunteering long term).

Areas that might have been given more attention include consideration of different models of volunteering and partnership, the need to integrate mental health into existing primary care (where most patients are encountered) and mental health in secondary care (expanding specialist mental health workers is a barely feasible prospect for many countries, hence task-sharing across existing staff may be the best approach). Refugees and the linked issues of escalating conflict and climate change are of particular importance as the World Health Organization states that mental health disorders occur at twice the prevalence found in background communities. Despite numerous country examples, some of particular relevance have not been mentioned (such as Uganda – where I and at least one of the authors have significant experience – and Ukraine, Ethiopia and Palestine – where the Royal College of Psychiatrists has been involved in recent years). And some of the countries described are extremely dangerous to try to work in (e.g. Myanmar) and surely need more detailed on advice on how to contribute effectively with minimal danger to self and colleagues.

A minor quibble is that, especially since the recent COVID-19 pandemic, virtual platforms have significantly enhanced communications even for the most remote locations. Effective training and support are often possible at negligible financial and carbon emissions cost. And the many options now available are surely an improvement over pre-pandemic Skype?

However, the crucial point is that this book is a practical guide and to remain valuable it needs to be portable. So, the trade-off seems worth it overall. Mental disorders account for 14% of preventable deaths globally and the poorest countries have the highest rates and fewest resources to manage this. Volunteering in well-managed and sustainable programmes is a valuable part of tackling this unmet need and the opportunities that arise. The recent pandemic showed what a low priority all the world's national health systems accorded mental health – rich and poor alike. The consequences will take years to address. So, this book should be packed by volunteers along with Vikram Patel & Charlotte Hanlon's *Where There Is No Psychiatrist* and Lord Nigel Crisp's *Turning the World Upside Down Again*. But please read it *before* you set off.

